# Comparison of Stand-Alone Cage versus Intervertebral Cage with Pedicle Screw and Rod Fixation in Dogs with Degenerative Lumbosacral Stenosis

**DOI:** 10.1055/a-2685-8054

**Published:** 2025-09-01

**Authors:** Eline J.C. van den Brink, Seyed A. Kamali, Anna R. Tellegen, Björn P. Meij

**Affiliations:** 1 Department of Clinical Sciences, Faculty of Veterinary Medicine, Utrecht University, Utrecht, The Netherlands

**Keywords:** dog, lumbosacral stenosis, pedicle screw and rod fixation, lumbosacral fusion

## Abstract

**Objective:**

The aim of this study was to assess the clinical outcome of treatment of degenerative lumbosacral stenosis in dogs with a stand-alone intervertebral spacer (S group) and combined with a pedicle screw and rod fixation (S + PSRF group) in the lumbosacral junction.

**Study Design:**

Retrospective study. Medical records (2014–2023) were reviewed for dogs treated with a stand-alone intervertebral spacer (S group) or a spacer combined with PSRF (S + PSRF group). Data collected included clinical signs at the time of presentation, surgical technique, implant type, perioperative bacterial culture, complications, outcomes and subsidence.

**Results:**

Minor complications occurred in 10/11 dogs in the S group and 6/17 dogs in the S + PSRF group. Major complications occurred in 3/11 dogs in the S group and 5/17 dogs in the S + PSRF group. Long-term outcome was excellent in 63.6% dogs in the S group and 64.7% dogs in the S + PSRF group. Subsidence was noted in 75% of the cases in the S group compared with 33% of cases in the S + PSRF group. Bacterial cultures were positive in 6/28 cases.

**Conclusion:**

Both treatment options were associated with full return of function in 64 to 65% of cases. Complications were more frequent in the S group. The S + PSRF group showed less subsidence of the cage. There was more frequent evidence of bone ingrowth in the intervertebral spacer in the S + PSRF group. Based on the observations in this study, both treatment options are viable for the treatment of degenerative lumbosacral stenosis with similar long-term outcomes; however, S + PSRF may result in less subsidence and better fusion and may therefore be preferable.

## Introduction

Degenerative lumbosacral stenosis in middle-aged large breed dogs is characterized by degeneration of the L7–S1 intervertebral disc, narrowing of the intervertebral disc space, disc herniation, foraminal stenosis and proliferation of soft tissue and bone. 1 Dorsal laminectomy and microdiscectomy result in clinical improvement of 66.7 to 96.5% of dogs, but recurrence has been reported in up to 18% of treated dogs. 2 3 4 Instability of the L7–S1 intervertebral junction, further loss of disc height and foraminal stenosis will lead to an increase in degenerative changes with risk of damaging the nerve roots of the cauda equina. 5 6 In addition to decompression, distraction–fixation of the lumbosacral junction is advised when dynamic instability, disc height loss and foraminal stenosis are diagnosed. 1

Distraction restores foraminal width and disc height, relieving pressure on nerve roots exiting through the foramina. 7 Biomechanical evaluation of dorsal fixation with a pedicle screw and rod fixation (PSRF) following dorsal laminectomy and discectomy has shown increased stability of the lumbosacral joint. 8 From a biomechanical standpoint, the ventral vertebral column is not addressed in PSRF alone. 9 The use of an intervertebral spacer (S), like a cage, restores stability to a state comparable with the native spine, with the benefit of restoration of the disc height and foraminal width. 9 The use of a spacer in combination with PSRF (S + PSRF) addresses both the dorsal and ventral vertebral column and results in a more rigid fixation and might reduce the risk of cage migration. 9 These methods of fixation have not yet been compared in a clinical setting, and short- and long-term outcomes have not been assessed for these methods of fixation.

The aim of this study was to compare dogs treated with distraction–fusion of the lumbosacral joint with a stand-alone cage and with S + PSRF by reporting complications, clinical outcome and subsidence of the spacer to determine if the combination produces a better clinical outcome than the spacer alone. We hypothesize that S + PSRF leads to a more rigid fixation of the L7–S1, therefore, leading to a better clinical outcome.

## Materials and Methods

### Patient Selection

Medical records of dogs with degenerative lumbosacral stenosis in the Small Animal Clinic at Utrecht University were retrieved from January 2014 to June 2023, which were treated with an intervertebral spacer between L7 and S1 with or without the addition of PSRF fixation. Medical records were evaluated for preoperative clinical signs, diagnosis, surgical technique, implant type, perioperative bacterial culture, complications, pre- and postoperative imaging and clinical outcome. Dogs were included if the diagnosis of degenerative lumbosacral stenosis was confirmed by imaging (magnetic resonance imaging/computed tomography), and if there was no evidence of imaging, then signs of discospondylitis on advanced imaging. In addition, a minimal follow-up time of 6 weeks, including imaging, was required for inclusion. Dogs were excluded if they had incomplete medical records, lacked the minimal follow-up time of 6 weeks or had concurrent lumbosacral disease other than degenerative lumbosacral stenosis. Dogs were scored a 1 for mild clinical signs such as reluctance to walk or jump with pain on deep palpation of the LS junction, a 2 for moderate clinical signs such as lameness, nerve root signature and pain on palpation of the LS junction and a 3 for clinical signs combined with neurological deficits ( Supplementary Tables 1 and 2 , available in the online version only). Median age and body weight were calculated, and the chi-square test of independence was used to determine statistical differences in patient sex and neuter status.

### Surgical Technique

All surgeries were performed by the same surgeon, and the surgery included dorsal laminectomy, discectomy and stabilization. When dogs had received prior surgery, the previous dorsal laminectomy was revised and enlarged if needed. A standard anaesthesia protocol was used, starting with an intramuscular injection of methadone (0.2 mg/kg), dexmedetomidine (5 μg/kg) and ketamine (0.5 mg/kg), followed by intravenous induction with propofol (1–4 mg/kg). Anaesthesia was maintained with isoflurane and constant rate infusion of ketamine (5–10 μg/kg/min) and/or dexmedetomidine (0.5–2 μm/kg/h) as needed. Cefazoline (20 mg/kg) was given intravenously as a perioperative antibiotic medication at least 30 minutes before incision and repeated every 90 minutes. The intervertebral disc space was prepared by adapting the discectomy to the size of the cage and curettage of the cartilaginous endplates using a small curette. A sample was taken from the intervertebral disc space for bacterial culture. Dogs received either a titanium cage (SynCage-C short implant, curved cage, width 15 mm, depth 12.5 mm, height 4.5 mm; 495.221S, DePuy Synthes, Amersfoort, the Netherlands) or a three-dimensionally printed titanium cage with similar or smaller dimensions (Rita Leibinger, GmbH & Co., Muehlheim, Germany; Supplementary Tables 1 and 2 [available in the online version only]). The surgical procedure for cage placement was performed as described by Teunissen and colleagues. 9 Correct placement of the cage was verified using intraoperative fluoroscopy.

**Fig. 1:**
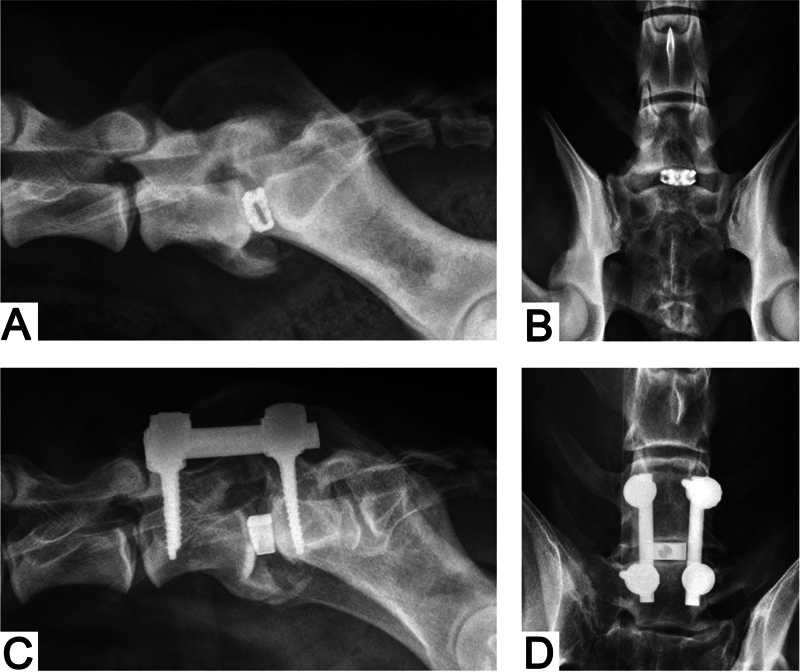
Postoperative lateral ( **A, C** ) and ventrodorsal ( **B, D** ) radiographs of a dog with degenerative lumbosacral stenosis treated with a spacer ( **A, B** ; spacer group) and a dog treated with a spacer and pedicle screw and rod fixation, there is a transitional S1–S2 present in this dog ( **C, D** ; spacer and pedicle screw and rod fixation group).

The second patient group (S + PSRF) received, in addition to the cage, fixation with four monoaxial titanium pedicle screws (length 25 mm, diameter 4.0 mm) and two 6-mm titanium rods (DePuy Synthes) or fixation with four polyaxial titanium pedicle screws (length 24 mm, diameter 3.5 mm) with two 3.5-mm titanium rods (Travmavet, Ankara, Turkey; Supplementary Table 2 [available in the online version only]). Placement of the pedicle screws was performed as described by Smolders and colleagues, and correct placement was verified by intraoperative fluoroscopy in both groups. 5 All dogs received a local morphine splash block (0.1 mg/kg), followed by placement of a fat graft dorsal to the cauda equina. After routine closure, postoperative radiographs were made ( Fig. 1 ). Dogs were hospitalized for recovery and pain medication with methadone (0.1–0.2 mg/kg, six times a day) and ketamine constant rate infusion (3–5 μg/min). Dogs were discharged 1 to 2 days after surgery with a 2-week course of nonsteroidal anti-inflammatory drugs (meloxicam 0.1 mg/kg, *per os* , once a day or carprofen 2 mg/kg, *per os* , twice a day) and gabapentin (10 mg/kg, *per os* , thrice a day) and in 17/30 cases with 7 days of amoxicillin and clavulanic acid (12.5 mg/kg, *per os* , twice a day).

### Follow-Up

All dogs received 6 weeks of recommended leash restraint with a 6-week rehabilitation schedule; physiotherapy was encouraged starting 2 weeks after surgery. Dogs were invited for a 6-week follow-up appointment, including radiographic examination of implant positioning, which was considered a short-term follow-up. Medium follow-up appointments were recommended at 3 and 6 months, including imaging by radiography or CT to confirm spinal fusion, but were left to the owner's willingness to return to the clinic. Information on long-term follow-up was collected by return visits requested by the owner or by telephone follow-up and questionnaires. Follow-up times varied from 6 weeks to 5 years. Complications were defined as minor when surgical intervention was not required; however, additional medical management was provided, such as an extended course of oral analgesia. The need for additional pain medication was considered a complication when patients were unable to discontinue oral pain medication after the standard prescription duration and needed an extension of at least 1 week of oral pain medication. Rehospitalization due to ongoing pain was considered a major complication, but did not occur in our patient cohort. Clinical outcome was assessed by the absence of pain and lameness during follow-up appointment and scored as 1: Poor when additional surgery was needed or the patient was euthanatized due to relapse; 2: Fair when patients were comfortable on additional pain medication; or 3: Good when a complete recovery was recorded. Supplementary Table 1 (available in the online version only) provides an overview of the different cases in the S group, and Supplementary Table 2 (available in the online version only) provides an overview of the different cases in the S + PSRF group with outcome and follow-up times.

### Radiographic Evaluation

**Fig. 2:**
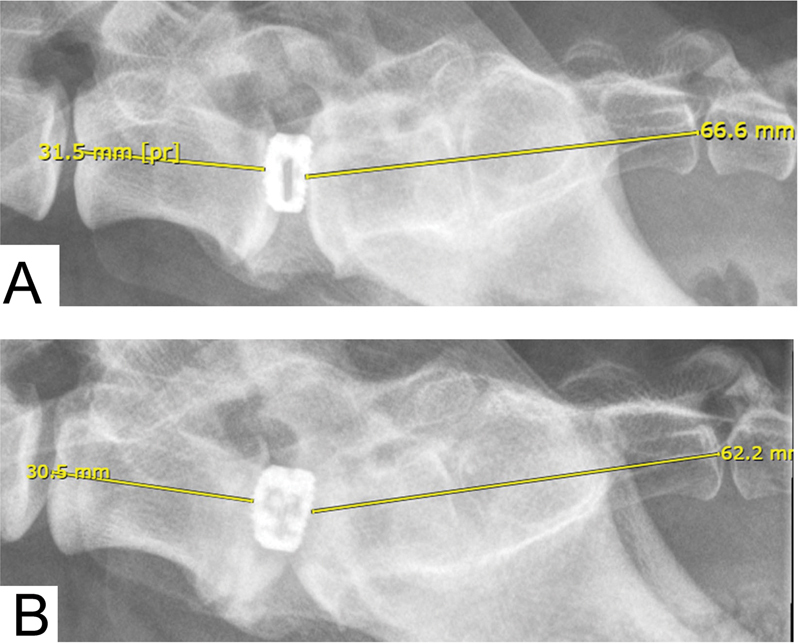
Measurement of vertebral body length of L7 and S1–S3 on the postoperative radiograph after placement of a stand-alone intervertebral spacer ( **A** ). The subsidence of the cage at 12 weeks after surgery was 7% at the caudal end plate of L7 and 3% at the cranial end plate of S1 ( **B** ).

Radiographs and computed tomography images obtained at 6 or 12 weeks postoperative were compared with immediate postoperative radiographs for subsidence of the implant, implant positioning and the development of adjacent segmental disease. Subsidence during follow-up was measured as a percentage decline of the vertebral body length of L7 and S1–S3. Vertebral body length was measured from the middle of the cranial (L7) or caudal (S1–S3) end plate to the middle of the cranial (L7) or caudal (S1–S3) contact surface of the intervertebral cage ( Fig. 2 ). Computed tomography images at a minimum of 3 months postoperative were evaluated for signs of bone fusion between the L7 and the S1 vertebral bodies through the intervertebral cage.

### Histological Analysis

The treated level of one dog for each treatment approach (S and S-PRSF) became available for postmortem analysis and was embedded into polymethyl methacrylate for histological examination. The harvested samples were first dehydrated using a dilution of ethanol solutions. Following, the specimens were immersed in a plastic embedding solution (made up of polymethyl methacrylate monomers and initiators) in a desiccator to achieve dehydration and optimum penetration. The polymethyl methacrylate-infiltrated specimens were carefully placed into moulds, followed by regulated temperature and curing time to induce polymerization. The specimen was subsequently sectioned to 70- to 80-μm thickness using a saw microtome (Leica SP 1600, Wetzlar, Germany). All sections were subsequently stained with methylene blue and acid fuchsin and examined under light microscopy (Olympus BX53, Tokyo, Japan).

## Results

### Demographics

The study included 28 dogs; 11 dogs were treated with an S (S group), and 17 dogs also received a PSRF (S + PSRF group). The following breeds were represented: 10 German Shepherds, 4 Labrador Retrievers, 3 Rhodesian Ridgebacks and 1 of each of the following breeds: American Bulldog, Czechoslovakian Vlcak, Slovenský Hrubosrstý Stavač, Weimaraner, Airedale Terrier, Leonberger, Golden Retriever, Bouvier, Belgian Shepherd, French Bulldog, Český Fousek and Border Collie. The median age in both groups was 7 years, S group; 1 to 13 years, S + PSRF group; 2 to 10 years. The median body weight in the S group was 38.7 kg (28.8–55.6 kg) and in the S + PSRF group 38.4 kg (15.3–65.6 kg). There was one entire female, three neutered females, four entire males and three neutered males present in the S group; there was one entire female, four neutered females, seven entire males and five neutered males present in the S + PSRF group. There was no significant difference between sex and neuter status between groups, *p* -value of 0.941. None of the patients in the S group and 4/17 patients in the S + PSRF group had previous surgery to the L7–S1 junction ( Supplementary Tables 1 and 2 , available in the online version only).

### Clinical Signs

Prior to surgery, all dogs showed signs of reluctance to walk or pain when jumping or standing up. Dogs showed clear pain with deep palpation of the L7–S1. In 16/28 dogs, unilateral lameness was noted; in 7/28 dogs, dragging of the hindlimbs was noticed. Spinal reflexes were normal in 22/28 dogs; (pseudo)hyperreflexia of the patellar tendon reflex was noted in 6 dogs. All dogs showed normal postural reactions. Clinical signs were graded as 2 in 7/11 cases and grade 3 in 4/11 cases in the S group. Clinical signs were graded as 2 in 13/17 cases and grade 3 in 4/17 cases in the S + PSRF group ( Supplementary Tables 1 and 2 , available in the online version only). There was no difference in patient presentation between groups.

### Bacterial Culture

Positive bacterial cultures occurred in 6/28 cases. In all cases, bacteria were identified after accumulation and prolonged incubation of the samples with very low bacterial counts. The following bacterial species were identified: *Kocuria* spp., *Staphylococcus pseudointermedius* , *Dermacoccus nishinomiyaensis* , *Salmonella* spp., *Brevibacterium casei* and *Deinococcus wulumuqiensis* . Culture results did not influence antibiotic medication treatment.

### Complications

Minor complications occurred in 10/11 dogs in the S group and consisted of the need for additional pain medication (5/11), temporary neuropraxia of the tail and/or hindlimbs (1/11) and temporary atonia of the bladder and/or rectum (5/11). Major complications occurred in 3/11 dogs and consisted of dorsal displacement of the cage in two dogs and in sudden relapse 6 weeks postoperative, leading to euthanasia in one dog. In both dogs with dorsal displacement of the cage, revision surgery was performed, and the cage was replaced, and dorsal stabilization was added by PSRF of the L7–S1 junction.

**Fig. 3:**
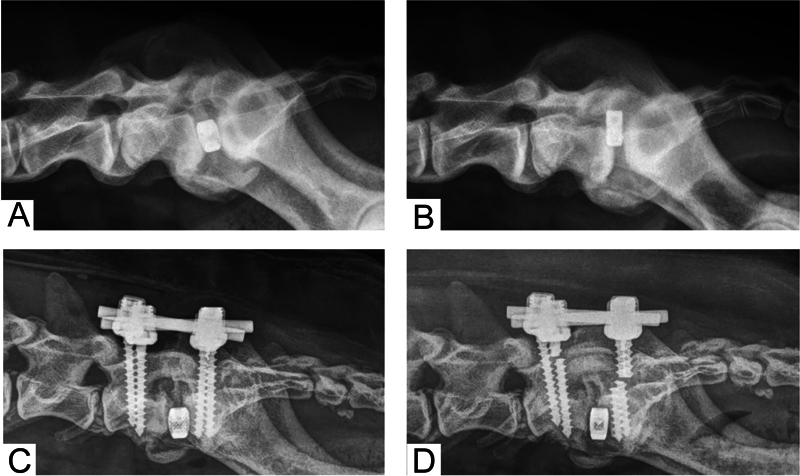
Postoperative radiographs of a dog treated with a stand-alone cage ( **A** ). Dorsal displacement of the cage was seen at 10 weeks postoperatively ( **B** ). Postoperative radiographs of a dog treated with an intervertebral spacer and pedicle screw and rod fixation ( **C** ). Implant failure was seen 6 weeks postoperatively ( **D** ), and three of the four polyaxial pedicle screws were broken.

Minor complications occurred in 6/17 dogs in the S + PSRF group and consisted of need for additional pain medication (3/17), temporary neuropraxia of the tail and/or hindlimbs (2/17) and implant failure by broken pedicle screws not needing revision surgery (1/17; Fig. 3 ). Major complications occurred in 5/17 dogs and consisted of relapse of clinical signs at 10 weeks and 3 months in 2 dogs leading to euthanasia, aspiration pneumonia and discospondylitis in 1 dog (1/17), dorsal displacement of the cage or broken pedicle screws needing revision surgery in 2 dogs ( Fig. 3 and Supplementary Table 2 [available in the online version only]). Both dogs underwent revision surgery; in the first case, the cage was removed and the PSRF was maintained. In the second case, the PSRF was removed, and the cage was replaced and left without additional dorsal fixation of the L7–S1 junction.

### Outcome

In the S group, two dogs had revision surgery, and one dog was euthanatized 6 weeks postoperative due to relapse of neurological signs and lumbosacral pain after initial uneventful recovery, scoring a 1 for outcome. Follow-up in the remaining eight cases varied from 3 months to 5 years. One dog showed a relapse of clinical signs 2 years postoperative, which was managed conservatively until the dog died of unrelated causes 4 years postoperative. Because the overall quality of life with pain medication in this dog was good, this patient scored a 2 for outcome. The remaining seven dogs showed no signs of relapse and a return to full function, scoring a 3 for outcome.

In the S + PSRF group, two dogs underwent revision surgery, and two dogs were euthanatized because of relapse of clinical signs at 10 weeks and 3 months postoperative, scoring a 1 for outcome. All dogs but one showed initial clinical improvement.

Of the remaining 12 dogs, one dog showed a relapse in clinical signs 5 months postoperatively, which could be managed with pain medication. Because the overall quality of life with pain medication in this dog was good, this patient scored a 2 for outcome. All remaining 11 dogs showed no signs of relapse and a return to full function, scoring a 3 for outcome.

### Radiographic Evaluation: Subsidence and Adjacent Segment Disease

Preoperative imaging consisted of a magnetic resonance imaging scan for 10/11 dogs in the S group and 14/17 dogs in the S + PSRF group. In the remaining cases, a preoperative CT scan was performed. For all cases, immediate postoperative radiographs were available, and for 8/11 cases in the S group and all cases in the S + PSRF group, a radiograph at 6 weeks was available; in the other three dogs in the S group, a CT scan was performed at 6 weeks. Mid to long-term follow-up CT at 3 to 18 months was available for 4/11 cases in the S group and 4/17 cases in the S + PSRF group.

Images for measuring subsidence were available in 8/11 cases in the S group. Subsidence was noted at the caudal endplate of L7 and cranial endplate of S1 in 75% of cases in the S group. Subsidence of 3 to 9% was noted in the caudal endplate of L7, and subsidence of 2 to 16% was noted in the cranial endplate of S1.

Images for measuring subsidence were available in 14/17 cases in the S + PSRF group. Subsidence was noted at the caudal endplate of L7 and the cranial endplate of S1 in 33% of cases. Subsidence of 3 to 8% was noted in the caudal endplate of L7, and subsidence of 2 to 4% was noted in the cranial endplate of S1. One dog showed radiographic signs of adjacent segment degeneration at the L6–L7 junction on radiographs 2 years post-S + PSRF without clinical signs.

**Fig. 4:**
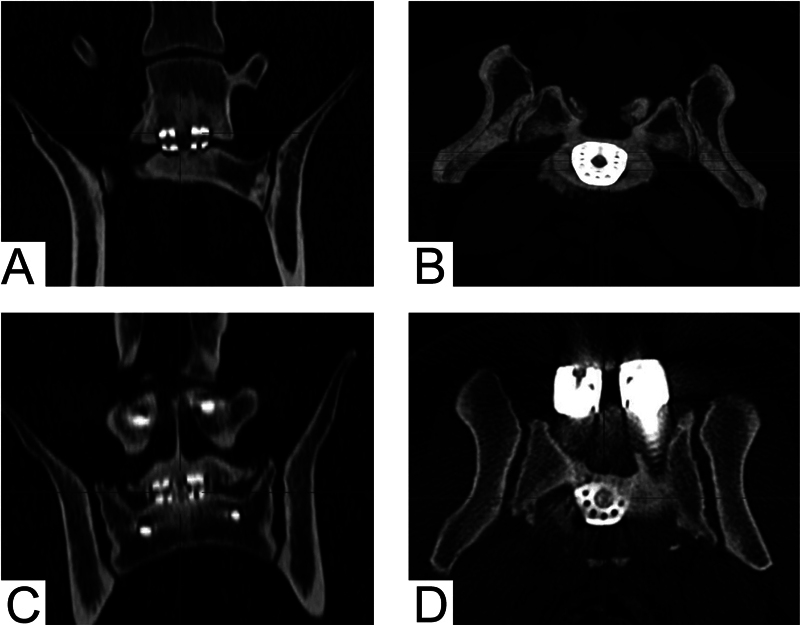
Axial ( **A, C** ) and transverse ( **B, D** ) images of computed tomographic scan 3 months postoperatively of a dog treated with a stand-alone cage ( **A, B** ; spacer group) and a dog treated with a cage and pedicle screw and rod fixation ( **C, D** ; spacer and pedicle screw and rod fixation group). Bone ingrowth was present inside the cage in the spacer and pedicle screw and rod fixation group ( **C, D** ), but not in the spacer group ( **A, B** ).

Computed tomography images taken at least 3 months postoperatively were available for 4/11 cases in the S group and 4/17 cases in the S + PRSF group. In the S group, only 1/4 dog showed signs of L7–S1 fusion through the intervertebral spacer, whereas 4/4 dogs in the S + PRSF group showed signs of L7–S1 fusion through the spacer ( Fig. 4 ).

### Histological Examination

**Fig. 5:**
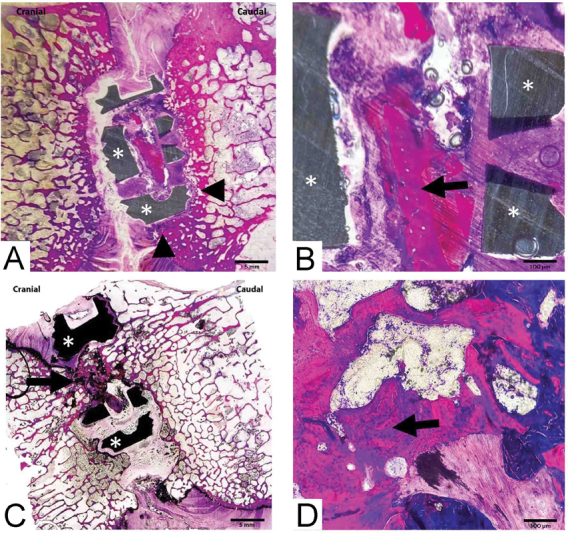
Postmortem histology sections of the L7–S1 region stained with methylene blue and acid fuchsin in two dogs with degenerative lumbosacral stenosis treated with a stand-alone cage ( **A, B** ; spacer group, 1.5 years postoperatively) and a cage and pedicle–screw rod fixation ( **C, D** ; spacer and pedicle screw and rod fixation group, 2 years postoperatively). In the spacer group ( **A, B** ), the cage (*) was predominantly filled with fibrous connective tissue and some new bone formation (arrow), but spinal fusion from end plate to end plate was not confirmed. Moreover, cage subsidence was noted in the caudal endplate (arrowheads). In the spacer and pedicle screw and rod fixation group ( **C, D** ), bone bridging (arrow) through the cage (*) was continuous from end plate to end plate, confirming spinal fusion. Source for ( **C** ): Adapted from Reints Bok et al. 10 Licensed under CC BY 4.0.

Postmortem specimens of two dogs were available for histological examination and assessment of bone ingrowth through the intervertebral cage. The histopathology of the S + PSRF case showed evidence of bone fusion between L7 and S1 through the intervertebral cage ( Fig. 5 ). In contrast, the micrographs of the S group case showed significant subsidence of the implanted cage within the S1 vertebral bone. While new bone formation was also observed inside the cage, spinal fusion was not confirmed as the treated level was predominantly filled by fibrous connective tissue ( Fig. 5 ). 10

## Discussion

There was no clear difference in long-term clinical outcome between the S and S + PSRF groups, and both treatment options were associated with full return of function in 64 to 65% of cases. Complications were more frequent in the S group, and minor complications in this group were mainly related to the insertion of the cage. Lateral retraction of the cauda equina to enable insertion of the cage was the most likely cause of tail and bladder dysfunction seen shortly after surgery. The S + PSRF group showed less subsidence of the cage; the frequency of dorsal migration of the cage was similar in both groups. Major complications were similar in both groups and were mainly associated with a sudden relapse in pain. Most dogs were evaluated and euthanatized by the referral veterinarian, and the exact cause of the relapse of symptoms is not known in these cases.

Subsidence was more frequently observed in the S group. From a biomechanical point of view, this can be explained by ongoing instability due to a lack of rigid dorsal fixation between L7 and S1 after placing a stand-alone cage, which only addresses the ventral spinal column. 9 Micromovement in the intervertebral disc space between the cage and the endplates might result in trauma of the endplates, resulting in subsidence. The linear compression force will only be countered by the cage, whereas in the S + PSRF group, forces will be countered by the cage and the PSRF, resulting in more force on the end plates in the S group compared with the S + PSRF group. 11 Although subsidence of the implants was observed in both S and S + PSRF groups, there was no association between subsidence noted on radiographic examination and clinical outcome in our patient group. In human literature, subsidence is noted in up to 40% of patients after placement of an intervertebral cage. Subsidence does lead to recurrent narrowing of the intervertebral foramina but does not seem to influence clinical outcome, which is consistent with our findings. 12 13

Adjacent segment disease is considered a complication in human medicine, and adjacent segment degeneration is noted in 26% of lumbar fusion patients. 14 The cadaveric study of Zindl and colleagues described an increased motion of the L6–L7 junction after placement of an intervertebral bolt and pedicle screw and rod system. In our patient cohort, there was one dog with radiographic evidence of adjacent segment degeneration, without recurrence of clinical signs. 15 The low prevalence found in our study could be due to the low patient number, lack of long-term follow-up, shorter life span in dogs or because of different kinematic loading of the canine spine, rendering our canine patients less prone to developing adjacent segment disease despite increased motion of the L6–L7 junction after stabilization of the L7–S1 junction.

The number of minor complications was higher in the S group compared with the S + PSRF group. More dogs were in need of prolonged pain medication in the S group compared with the S + PSRF group, and dogs experienced postoperative neuropraxia of the cauda equina more often. This could be due to instability of the L7–S1 junction, leading to ongoing pain and damage of the cauda equina. Displacement of the cage was observed in one dog in both groups. Displacement in the S group is considered to be the result of ongoing movement and instability at the lumbosacral junction. The dog that showed displacement in the S + PSRF group had a relatively small cage inserted compared with the height of the intervertebral disc, and the small foot plate may have acted as a stress riser. Possibly, the small cage was not sufficiently fixed by PSRF between the end plates, resulting in space for the cage to migrate dorsally. One of two dogs in the S + PSRF group treated with the polyaxial pedicle screw system showed failure of the screws. It is hypothesized that there will be less force on the dorsal PSRF construct when combined with a spacer because part of the distraction force between the vertebral bodies will be neutralized by the cage, leading to less strain on the pedicle screws. According to Chen and colleagues and Wang and colleagues, there is more stability of the spine when stabilized with monoaxial pedicle screws versus polyaxial pedicle screws. 16 17 Continued loading in combination with a smaller diameter may have been a factor in implant failure in this dog.

### Bacterial Cultures

None of the dogs with a positive culture at the time of surgery developed clinically active discospondylitis. In human medicine, cultures of intervertebral disc spaces are positive in 9 to 53% of samples. 18 Most samples are positive for *Cutibacterium acnes* ; some authors suggest a higher incidence of positive cultures in diseased discs, but this bacterium is also cultured from healthy disc material. 18 19 In the positive cultures of our patient groups, no single identical bacterium was found, and bacterial counts were very low. Contamination of the samples leading to false positive cultures must be considered. Only two of the samples were positive for bacteria, which are described as pathogenic in earlier studies. 3 The prevalence of bacteria in healthy discs in dogs is unknown. Based on the current findings, there is no need for additional routine postoperative treatment with antibiotic medications.

### Limitations

The retrospective nature of the study resulted in a lack of standardized data. Both groups contained only a small number of dogs that were not randomly assigned to either the S or S + PSRF group, limiting the ability to perform a statistical analysis. Patient positioning for radiographic examination was not standardized. Patient positioning might be of influence when measuring subsidence. Measurements based on CT can mitigate this limitation. There were multiple factors influencing the decision to perform S or S + PSRF in a dog. The cost of adding PSRF to an intervertebral cage may have influenced the owner's decisions as to which operation was performed. Because of the smaller patient number in the S group compared with the S + PSRF group, the short-term complications and long-term outcome could be over- or underestimated.

## Conclusion

There was no clear difference in long-term clinical outcome between the S and S + PSRF groups, and both treatment options were associated with full return of function in 64 to 65% of cases. Short-term complications were more frequent in the S group and were mainly related to the cage insertion. The S + PSRF group showed less subsidence of the cage. There was more frequent evidence of bone ingrowth on CT and histological analysis in the S + PRSF group, compared with the S group. Based on the preliminary results of the present study, both treatment options are viable for the treatment of degenerative lumbosacral stenosis; however, S + PSRF may result in less subsidence and better fusion and may therefore be preferable. As described by Kurkowska and colleagues decision for the best treatment should be guided by patient-specific anatomical and pathological changes. 20 A larger prospective study with random allocation to the S or S + PSRF group and standardized follow-up is needed to make a final recommendation about the best distraction–fixation treatment for degenerative lumbosacral stenosis.
