# Supplementary material for: Comparison of Stand-Alone Cage versus Intervertebral Cage with Pedicle Screw and Rod Fixation in Dogs with Degenerative Lumbosacral Stenosis
Source: Vet Comp Orthop Traumatol. 2025 Sep 1;39(3):169–76. doi: 10.1055/a-2685-8054 (PMC13288431; doi:10.1055/a-2685-8054)
Supplement: Supplementary file 1 — Supplementary Material [file 10-1055-a-2685-8054-s24090078.pdf]

Supplementary Table 1. Details of dogs with degenerative lumbosacral stenosis that underwent treatment with a stand-alone intervertebral spacer (S).

NR	BREED	AGE/SEX	BW(KG)	PREVIOUS SURGERY	GRADE CLINICAL SIGNS[A]	TREATMENT	OUTCOME[B]	SUBSIDENCE %		MINOR COMPLICATIONS	MAJOR COMPLICATIONS	FOLLOW UP
								CRANIAL	CAUDAL			
1.	GSD	1yr M	36.2	No	3	S	1	NA	NA	Tail neuropraxia, bladder and rectal atonia	Relapse at 6 weeks, euthanasia	6w
2.	American Bulldog	4 yr MN	55.6	No	3	S	3	0	2	Additional pain medication		5yr
3.	GSD	7 yr MN	41.8	No	3	S	3	3	1.7	Additional pain medication		5yr
4.	Labrador retriever	6 yr F	28.8	No	2	S	2	0	0	Bladder atonia, relapse at 2yrs successful medical management		4yr
5.	Labrador retriever	7yr FN	37.4	No	2	S	1	NA	NA	Additional pain medication	Dorsal displacement of cage at 14 days needing revision	14 days
6.	Czechoslovakian Vlcak	13 yr MN	36.3	No	2	S	3	9	16.5	Additional pain medication		1.5yr
7.	Slovenský hrubosrstý stavač	8 yr FN	38.7	No	2	S	3	3	0	Rectal atonia		3yr
8.	Weimaraner	9 yr FN	29.0	No	3	S	3	4	2	None		3m
9.	GSD	6yr M	38.9	No	2	S	3	3	12	Bladder atonia		2yr
10.	GSD	4yr M	39.1	No	2	S	3	6	1.8	Additional pain medication		2yr
11.	Rhodesian ridgeback	7yr M	43.1	No	2	RL size L	1	NA	NA	Bladder atonia	Dorsal displacement of cage at 10 weeks needing revision	10 w

GSD= German shepherd dog, yr=year, M=male, MN=male neutered, F=female, FN=female neutered, S=syncage, RL=Rita Leibinger cage, size L= large, , NA= not available, w=weeks, m=months. [A] Clinical signs: 1=mild, 2=moderate, 3=severe with neurological signs. [B] Outcome: 1=poor, 2=fair, 3=good.

Supplementary Table 2. Details of dogs with degenerative lumbosacral stenosis that underwent treatment with a stand-alone intervertebral spacer and pedicle screw and rod fixation (S+PSRF).

NR	BREED	AGE/SEX	BW (KG)	PREVIOUS SURGERY	GRADE CLINICAL SIGNS[A]	TREATMENT	OUTCOME[B]	SUBSIDENCE %		MINOR COMPLICATIONS	MAJOR COMPLICATIONS	FOLLOW UP
								CRANIAL	CAUDAL			
1.	Airedale terrier	9yr M	34.8	Yes	2	S+DPS	2	8.3	4.5	Tail neuropraxia, implant failure not needing revision, discospondylitis at 2 months, relapse at 5 months with successful medical management	Aspiration pneumonia	5m
2.	Leonberger	2yr MN	65.6	No	3	S+DPS	3	0	0			2yr
3.	Golden retriever	10yr M	47.8	Yes	3	S+DPS	1	NA	NA		Relapse at 3 months, euthanasia	3m
4.	GSD	4yr FN	42.5	No	2	S+DPS	3	3.3	1.8			4.5yr
5.	Bouvier	5yr M	46.2	Yes	3	S+DPS	1	0	0	Additional pain medication	Remains painful po, euthanasia	10w
6.	Rhodesian ridgeback	10 yr MN	52.4	No	2	S+DPS	3	0	0	Additional pain medication		5yr
7.	GSD	6 yr F	32.7	No	2	S+DPS	3	3	2			8m
8.	GSD	7yr FN	42.9	No	2	S+DPS	3	0	0			3yr
9.	GSD	7yr MN	38.8	No	2	S+DPS	3	3	1.8			1yr
10.	Labrador retriever	8yr FN	33.4	Yes	2	S+DPS	2	8	2	Additional pain medication		4m
11.	Rhodesian ridgeback	10 yr MN	45.5	No	3	RL size L+DPS	1	NA	NA		Dorsal displacement of cage needing revision at 8 weeks	8w
12.	Belgian shepherd	8yr FN	26.7	No	2	RL size L + DPS	3	0	1			1.5yr
13.	French Bulldog	7yr MN	15.3	No	2	RL size S+TV	1	NA	NA		Implant failure at 6 weeks needing revision	6w
14.	Labrador retriever	7yr MN	38.4	No	2	RL size L +DPS	3	0	0			6m
15.	GSD	9yr M	37.5	No	2	RL size L+DPS	3	0	0			6m
16.	Český Fousek	6yr M	33.6	No	2	RL size XL+DPS	3	1	1	Tail neuropraxia		1yr
17.	Border Collie	9yr MN	19.8	No	2	RL size M+TV	3	1	1			5m

GSD= German shepherd dog, yr=year, M=male, MN=male neutered, F=female, FN=female neutered, S=syncage, DPS= monoaxial pedicle screw and rod system by DePuy Synthes, RL=Rita Leibinger cage, size S= small, size L= large, size XL= extra large, TV= polyaxial pedicle screw and rod system by Travmavet, , NA= not available, w=weeks, m=months. [A] Clinical signs: 1=mild, 2=moderate, 3=severe with neurological signs. [B] Outcome: 1=poor, 2=fair, 3=good.
